# Design and validation of a key performance indicator dashboard for equity in community and retail pharmacy services in Pakistan

**DOI:** 10.1371/journal.pone.0349972

**Published:** 2026-07-10

**Authors:** Muhammad Hammad, Rasikh Arif, Ali Zeb Khan, Ali Nasir

**Affiliations:** 1 Department of Community Health Sciences, Sohail University, Karachi, Pakistan; 2 Shifa College of Pharmaceutical Sciences, Shifa Tameer-e-Millat University, Islamabad, Pakistan; 3 School of Public Health, AI-Shifa Trust Eye Hospital, Rawalpindi, Pakistan; 4 Faculty of Pharmacy and Pharmaceutical Sciences, Ziauddin University, Karachi, Pakistan; SKYDA Health Nigeria, NIGERIA

## Abstract

**Background:**

Community and retail pharmacies are essential to primary healthcare delivery in Pakistan; however, significant disparities in workforce distribution, medicine availability, and service quality persist between urban and rural settings. The absence of standardized performance monitoring systems limits the ability to identify and address these inequities.

**Objective:**

To design, develop, and validate a key performance indicators (KPI) dashboard for monitoring equity in community and retail pharmacy service delivery in Pakistan.

**Methods:**

A multi-phase methodological study was conducted. Phase I involved qualitative needs assessment using semi-structured interviews with pharmacists, technicians, and administrators (n = 28) to identify service gaps. Phase II employed a modified Delphi technique with expert panel consensus to refine KPIs. Phase III included dashboard development using Microsoft Power BI. Phase IV comprised validation through content validity index (CVI) assessment and usability testing using the System Usability Scale (SUS). Quantitative data were analyzed using descriptive statistics, while qualitative data were analyzed thematically.

**Results:**

Eleven KPIs across workforce, efficiency, quality, safety, availability, and equity domains were finalized. Significant disparities were observed between urban and rural pharmacies, including higher stock-out duration (13.1 vs 6.2 days), lower counseling rates (55% vs 78%), and poorer workforce ratios (1:120 vs 1:65). The dashboard demonstrated high usability (SUS score: 82.5) and strong content validity (S-CVI/Ave: 0.89).

**Conclusion:**

The developed KPI dashboard provides a feasible and scalable tool for real-time monitoring of pharmacy performance and equity. Its implementation can support evidence-based decision-making and improve equitable pharmaceutical service delivery in Pakistan.

## Introduction

Community and retail pharmacies play a pivotal role in ensuring safe, effective, and accessible medication use within primary healthcare systems, particularly in low- and middle-income countries (LMICs) [1]. In regional and rural settings, these pharmacies serve as the primary point of contact for medication dispensing, patient counselling, and essential medicine supply management [2]. However, significant disparities in workforce distribution, medicine availability, and service quality persist between urban and rural community pharmacies, contributing to inequitable health outcomes and inconsistent pharmaceutical care delivery. The absence of standardized performance monitoring systems further limits the ability of health managers to identify gaps and implement targeted improvements [3].

Globally, Key Performance Indicators (KPIs) are widely used to evaluate healthcare service performance, including dimensions of efficiency, quality, safety, and access [4]. In pharmacy practice, KPIs commonly encompass medication availability, dispensing accuracy, counselling provision, and medication safety outcomes [5]. Several pharmacy KPI frameworks have been implemented internationally, including the Canadian Community Pharmacy Key Performance Indicators (cpKPIs) and quality indicator frameworks developed by the Pharmaceutical Care Network Europe (PCNE) [6–7]. These frameworks have demonstrated effectiveness in monitoring service quality and patient outcomes in high-income settings. While such frameworks have demonstrated utility in high-income settings, most existing models are not directly transferable to LMIC contexts, where rural pharmacies often face structural constraints such as workforce shortages, fragmented supply chains, limited digital infrastructure, and high patient demand [8]. Furthermore, many existing frameworks emphasize clinical and operational performance but inadequately capture geographic and equity-related disparities that are particularly relevant in resource-constrained settings [9–10]. These contextual differences necessitate the development of locally relevant, equity-focused performance measurement tools.

Equity is a central pillar of Universal Health Coverage (UHC), emphasizing fair access to essential health services regardless of geographic or socioeconomic status [11]. In Pakistan, rural populations frequently experience reduced access to qualified pharmacy personnel, higher medicine stock-out rates, and weaker pharmaceutical care infrastructure compared to urban settings [12]. National assessments have reported uneven distribution of registered pharmacists, lower regulatory compliance, and limited adoption of computerized pharmacy systems in rural and peripheral regions, further widening disparities in pharmaceutical service delivery [13–14]. These systemic inequities underscore the need for tools that not only measure pharmacy performance but also explicitly quantify disparities in service delivery across different healthcare settings.

Recent equity-focused implementation frameworks suggest that healthcare performance assessment should consider both service effectiveness and equitable distribution of healthcare resources. Gustafson et al. (2023) highlighted the importance of incorporating equity considerations into implementation frameworks to identify structural barriers and reduce healthcare disparities. Guided by this perspective, the present study conceptualized pharmacy performance across four domains: service accessibility, workforce capacity, medicine availability, and quality of pharmaceutical care. Together, these domains provide a framework for assessing equity in pharmacy service delivery across regional and rural settings [15].

Digital dashboards have emerged as effective tools for health system monitoring by integrating multiple indicators into visual, real-time platforms that support data-driven decision-making. In pharmacy practice, such dashboards can enhance transparency, facilitate performance tracking, and enable rapid identification of service gaps. Despite their growing adoption in hospital and health system management globally, there is a lack of standardized, equity-oriented pharmacy KPI dashboards designed specifically for community and retail pharmacy settings [16]. Moreover, currently available dashboard systems primarily focus on operational monitoring and often lack mechanisms to evaluate disparities in service delivery between different geographic and socioeconomic settings [17–18].

To address this gap, this study aimed to design, develop, and validate a community and retail pharmacy KPI dashboard to monitor service performance and assess equity in regional and rural settings in Pakistan. A rigorous multi-phase methodological approach was employed, including qualitative needs assessment, Delphi-based expert consensus for KPI refinement, dashboard development using Microsoft Power BI, and validation through the System Usability Scale (SUS) and content validity index (CVI) assessment.

By developing an evidence-based, equity-oriented performance monitoring tool, this study contributes to strengthening pharmacy management systems, improving accountability, and supporting equitable pharmaceutical care delivery. The resulting dashboard provides a practical mechanism for pharmacists, hospital administrators, and policymakers to identify service disparities, optimize resource allocation, and enhance decision-making within community and retail pharmacy systems in Pakistan.

## Study objectives

### Primary objective

To design and develop a validated community and retail pharmacy KPI dashboard for monitoring performance and assessing equity in service delivery across regional and rural settings in Pakistan.

### Secondary objectives

1. To identify key performance gaps and priority indicators through qualitative needs assessment in community and retail pharmacies.
2. To achieve structured expert consensus on essential pharmacy KPIs using a modified Delphi technique.
3. To develop a functional and user-friendly KPI dashboard using Microsoft Power BI.
4. To evaluate the usability of the dashboard using the System Usability Scale (SUS).
5. To assess content validity of selected KPIs using expert agreement and CVI analysis.
6. To propose a framework for potential integration of the dashboard into routine pharmacy management systems.

## Methodology

### Study design

This study employed a multi-phase methodological design to develop and validate a community pharmacy key performance indicators (KPI) dashboard aimed at assessing and improving equitable pharmaceutical service delivery across community and retail pharmacy settings in Pakistan. The methodological framework consisted of four sequential phases: (i) qualitative needs assessment to explore system-level gaps and equity-related challenges in community and retail pharmacy practice, (ii) KPI generation and prioritization using a modified Delphi consensus process, (iii) iterative design and development of a digital performance dashboard, and (iv) content validity assessment and usability testing of the final system. This mixed-methods developmental approach ensured methodological triangulation and enhanced both construct validity and practical applicability of the final tool.

### Setting

Study was conducted in community and retail pharmacy settings across Punjab, Pakistan, including both urban and rural locations from 20-April-2021–18- February 2024. Participants (n = 28) were purposively recruited from these settings to capture variability in infrastructure, staffing patterns, medication availability, patient volume, and operational workflows. This diversity provided a robust contextual foundation for identifying service delivery gaps and developing equity-oriented performance indicators for community and retail pharmacy practice. Participants were selected using purposive sampling to ensure inclusion of individuals with relevant expertise and experience in community and retail pharmacy settings. The final sample size was determined by thematic saturation, with interviews conducted until additional data ceased to yield novel themes or meaningful insights. Saturation was reached after 28 interviews, at which point data collection was concluded.

### Phase I: Needs assessment

#### Sampling and participants.

A purposive sampling strategy was used to recruit key stakeholders directly engaged in community and retail pharmacy service delivery. Potential participants were identified through professional pharmacy networks, local pharmacy associations, and institutional contacts across participating pharmacies. Eligible individuals were contacted via email or telephone and invited to participate. Of 34 individuals approached, 28 agreed to participate, yielding a response rate of 82.4%. Participants included community and retail pharmacists (n = 12), pharmacy technicians (n = 10), and pharmacy administrators (n = 6). Inclusion criteria required a minimum of one year of professional experience in community or retail pharmacy practice and active involvement in medication dispensing, inventory management, and patient counseling activities.

#### Data collection.

Data were collected through semi-structured, in-depth interviews designed to explore operational workflows, service delivery challenges, and equity-related disparities in community and retail pharmacy settings. Prior to formal data collection, the interview guide was pilot-tested with three pharmacists to evaluate the clarity, relevance, and comprehensiveness of the questions. Minor revisions were subsequently made to improve wording and ensure consistency in data collection. The final interview guide encompassed key domains including medicine availability, dispensing practices, staffing challenges, patient access barriers, workflow inefficiencies, and perceived differences in service provision between urban and rural pharmacies. Interviews were conducted either face-to-face or via secure online platforms according to participant preference and availability, with each session lasting approximately 20–30 minutes. All interviews were audio-recorded with prior informed consent and transcribed verbatim for analysis. Data collection and analysis were undertaken concurrently, allowing emerging findings to inform subsequent interviews. Recruitment continued until thematic saturation was achieved, defined as the point at which no new themes or meaningful insights emerged from additional interviews.

### Phase II: KPI selection using modified Delphi method

#### Expert panel.

A multidisciplinary expert panel comprising twelve members was formed for the Delphi process [19]. The panel included senior community pharmacists, academic pharmacy faculty, health systems researchers, and pharmacy administrators with oversight experience in community and retail pharmacy operations. This ensured contextual relevance and methodological rigor in KPI selection.

#### Delphi rounds.

A modified Delphi technique was conducted over two to three iterative rounds to achieve consensus on KPI selection [20]. In each round, experts evaluated proposed indicators based on relevance, feasibility, measurability, and importance in ensuring equitable pharmacy service delivery. A four-point Likert scale was used for scoring. Indicators achieving a mean score ≥3.5 and an item-level content validity index (I-CVI) ≥0.78 were retained. Feedback between rounds was summarized and used to refine and stabilize expert consensus.

The final KPI framework was categorized into four domains:

- **Accessibility** (i.e., pharmacist-to-patient ratio, medicine availability, patient waiting time)
- **Quality of care** (i.e., patient counseling rate, medication error reporting frequency)
- **Operational efficiency** (i.e., dispensing time, stock-out duration)
- **Equity** (i.e., rural–urban performance differentials, medicine availability disparity index)

### Phase III: Dashboard development

#### Software and tools.

Dashboard was developed using Microsoft Power BI as the primary analytics and visualization platform. Data integration was performed using Microsoft Excel and CSV datasets. Data transformation and KPI computation were executed using Data Analysis Expressions (DAX) to enable dynamic calculations and interactive reporting.

#### Dashboard architecture.

It consisted of a three-layer framework comprising (i) a data source layer, (ii) a data processing and analytics layer, and (iii) a visualization layer. Data source layer incorporated structured pharmacy datasets obtained from Excel and CSV files. Processing layer utilized Microsoft Power BI and Data Analysis Expressions (DAX) to perform data cleaning, transformation, KPI computation, and aggregation. The visualization layer generated interactive dashboards, KPI scorecards, trend analyses, and equity comparison modules. Relationships among datasets were established through a relational data model to enable automated KPI updates and real-time filtering across pharmacy settings, geographic locations, and reporting periods.

#### Dashboard features.

Dashboard incorporated interactive and real-time visualization features to support performance monitoring across community and retail pharmacy settings. Key features included color-coded performance indicators (red = poor, yellow = moderate, green = optimal), dynamic filtering by geography, pharmacy type, and time period, and multiple visualization formats such as bar charts, line graphs, and gauge indicators. The dashboard also included KPI summary cards, an equity comparison module, and automated alerts to highlight underperforming indicators requiring managerial attention.

#### Data model.

A structured relational data model was developed to support KPI computation. Key derived indicators included stock-out rate (proportion of days a medicine was unavailable), counseling coverage (proportion of patients receiving documented counseling), and disparity ratio (rural-to-urban KPI performance comparison). These measures enabled standardized and comparable assessment of performance across community and retail pharmacy settings.

#### Data verification.

To ensure data quality, operational records used for dashboard development were cross-checked against pharmacy inventory logs, dispensing registers, and administrative records where available. Where computerized pharmacy management systems were available, operational records were extracted directly from electronic databases. In pharmacies without computerized systems, data were obtained from paper-based dispensing and inventory records and subsequently verified through administrative review. Data completeness and consistency were reviewed before dashboard integration. Any discrepancies identified during verification were resolved through consultation with participating pharmacy personnel.

### Phase IV: Validation and usability testing

#### Content validity.

Content validity of the KPI framework and dashboard components was assessed through expert panel review. Item-level content validity index (I-CVI) and scale-level content validity index (S-CVI) were calculated to quantify agreement among experts. A threshold of ≥0.80 was considered acceptable, ensuring that retained indicators were methodologically robust and contextually appropriate for community and retail pharmacy practice.

#### Usability testing.

Usability evaluation was conducted among 20 end-users, comprising practicing community pharmacists and retail pharmacy administrators. Participants were recruited through purposive sampling to ensure representation of key stakeholder groups expected to use the dashboard in routine pharmacy practice. The System Usability Scale (SUS) was employed to assess perceived usability and ease of use of the dashboard. SUS scores were interpreted according to established benchmarks, with scores ≥80 indicating excellent usability, scores around 68 reflecting acceptable usability, and lower scores suggesting areas requiring further refinement.

#### Data analysis.

Data analysis was conducted using a structured, sequential approach aligned with the multi-phase design of the study. Initially, qualitative data from Phase I (needs assessment interviews conducted in community and retail pharmacy settings) were analyzed using Braun and Clarke’s six-step thematic analysis framework [21]. Two researchers independently reviewed and coded the interview transcripts, with discrepancies resolved through discussion and consensus. An iterative codebook was developed and refined throughout the analysis. Codes were subsequently grouped into broader themes reflecting operational challenges, service delivery gaps, and equity-related disparities in pharmacy practice. To enhance trustworthiness, investigator triangulation was employed, whereby a third researcher with expertise in pharmacy practice and qualitative methods reviewed the coding framework and emerging themes. Representative quotations were retained to support interpretation of the findings. The resulting themes informed the development of an initial pool of candidate key performance indicators (KPIs), which served as the foundation for the subsequent Delphi validation phase.

In Phase II, quantitative data obtained from the modified Delphi process were analyzed using descriptive statistical techniques. For each proposed KPI, measures of central tendency and dispersion, including mean scores and standard deviations, were calculated based on expert ratings. The level of expert agreement was assessed using the item-level content validity index (I-CVI) and scale-level content validity index (S-CVI). Predefined inclusion criteria (mean score ≥3.5 and I-CVI ≥ 0.78) were applied to determine the final set of KPIs. The degree of consensus across Delphi rounds was evaluated by examining changes in agreement scores and stability of responses between iterations.

In Phase III, operational pharmacy data from community and retail settings were cleaned, coded, and structured for integration into Microsoft Power BI. The operational data used during dashboard development and testing were derived from pilot pharmacy datasets collected from participating community and retail pharmacies and were not hypothetical datasets. Data transformation procedures were performed using Excel and Data Analysis Expressions (DAX) to compute standardized performance indicators. Key derived variables included stock-out rate, calculated as the proportion of days a medicine was unavailable; counseling coverage, defined as the proportion of patients receiving documented counseling; and other efficiency and accessibility metrics. These indicators were standardized to ensure comparability across diverse community and retail pharmacy settings.

In Phase IV, quantitative validation and usability data were analyzed using SPSS version 26. System Usability Scale (SUS) scores were computed according to established scoring guidelines and summarized using means and standard deviations to assess perceived usability and user satisfaction. Content validity indices (I-CVI and S-CVI) were re-evaluated at the final stage to confirm consistency of expert agreement on KPI relevance and dashboard structure. Descriptive analyses were also performed to examine equity-related indicators, including rural–urban disparity ratios in pharmacy service performance across community and retail settings.

Finally, qualitative feedback obtained during usability testing was analyzed using conventional content analysis to identify recurring themes related to usability, interpretability, workflow integration, and system feasibility. Quantitative and qualitative findings were triangulated to provide a comprehensive evaluation of the dashboard’s performance, ensuring methodological rigor and strengthening the validity, usability, and implementation readiness of the developed KPI system for community and retail pharmacy practice.

### Ethical considerations

Ethical approval for the study was obtained from ethics review committee of AI-Shifa Trust Eye Hospital Rawalpindi (Reference no: ERC-115/AST-21; Dated: 15-04-2021) prior to data collection. Written informed consent was obtained from all participants involved in interviews and usability testing. No patient-level or identifiable clinical data were collected during the study. All collected data were stored securely, and confidentiality of participants was strictly maintained throughout the research process.

## Results

### Participant characteristics

A total of 28 participants were included in the qualitative needs assessment phase, comprising community pharmacists (42.9%), pharmacy technicians (35.7%), and pharmacy administrators (21.4%). Participants were equally distributed across urban and rural community pharmacy settings (50.0% each), ensuring balanced representation of diverse practice environments. The mean years of professional experience varied across groups, with administrators demonstrating the highest experience (10.5 ± 3.4 years), followed by pharmacists (6.8 ± 2.1 years) and technicians (4.3 ± 1.9 years). These characteristics reflect a multidisciplinary and experienced sample suitable for identifying system-level challenges in pharmacy practice as shown in Table 1.

**Table 1:** Characteristics of participants in the needs assessment (N = 28).

Variable	Category	n (%)
**Profession**	Community Pharmacists	12 (42.9%)
	Pharmacy Technicians	10 (35.7%)
	Pharmacy Administrators	6 (21.4%)
**Practice Setting**	Urban Community Pharmacies	14 (50.0%)
	Rural Community Pharmacies	14 (50.0%)
**Mean Experience (years)**	Pharmacists	6.8 ± 2.1
	Technicians	4.3 ± 1.9
	Administrators	10.5 ± 3.4

### Needs assessment findings

Thematic analysis identified four major domains influencing pharmacy service delivery: workforce capacity and access, medicine availability, quality of service delivery, and operational efficiency. Workforce-related challenges included staffing shortages, unequal distribution favoring urban settings, and increased workload in rural pharmacies. Medicine availability issues were characterized by frequent stock-outs, supply chain delays, and budget constraints, particularly in rural settings.

Quality-related concerns included inadequate patient counselling, poor documentation practices, and lack of follow-up systems. Operational inefficiencies were reflected in longer dispensing times, workflow bottlenecks, and the absence of digital systems. These findings highlight systemic disparities between urban and rural community pharmacies and informed the initial development of candidate KPIs as mentioned in Table 2.

**Table 2:** Themes and subthemes identified in needs assessment.

Theme	Subthemes	Description
**Workforce Capacity and Access**	Staffing shortages	Limited pharmacist availability in rural pharmacies
	Unequal distribution	Workforce concentrated in urban settings
	High workload	Increased patient load in rural pharmacies
**Medicine Availability**	Stock-out frequency	Frequent shortages in rural retail/community pharmacies
	Supply chain delays	Procurement inefficiencies
	Budget limitations	Restricted procurement of essential medicines
**Quality of Service Delivery**	Inadequate counselling	Lower counselling rates in rural settings
	Poor documentation	Inconsistent manual records
	Lack of follow-up	No structured patient monitoring
**Operational Efficiency**	Long dispensing time	Slower dispensing in rural pharmacies
	Workflow bottlenecks	Crowding and manual processes
	Lack of digital systems	Absence of automation tools

### Delphi round 1: Initial KPI screening

In the first Delphi round, 29 candidate KPIs were evaluated across six domains. Key indicators such as pharmacist-to-patient ratio (mean = 4.6, I-CVI = 0.92), stock-out frequency (mean = 4.5, I-CVI = 0.96), and stock-out duration (mean = 4.4, I-CVI = 0.92) demonstrated strong consensus and were retained for further evaluation. Medication error reporting showed borderline agreement (I-CVI = 0.78) but was retained due to its relevance to patient safety as shown in Table 3.

**Table 3:** KPI ratings from Delphi Round 1 (initial screening, N = 29 KPIs).

KPI Category	KPIs (Round 1)	Mean Rating	I-CVI	Decision
**Workforce**	Pharmacist-to-patient ratio	4.6	0.92	Retain for Round 2
	Workforce equity ratio	4.3	0.83	Retain
**Efficiency**	Average dispensing time	4.1	0.88	Retain
	Patient waiting time	3.9	0.81	Retain
**Quality**	Counseling rate (%)	4.2	0.83	Retain
	Documentation completeness	4.0	0.80	Retain
**Safety**	Medication error reporting	3.7	0.78	Borderline retain
**Availability**	Stock-out frequency	4.5	0.96	Retain
	Stock-out duration	4.4	0.92	Retain
	Essential medicine availability	4.3	0.89	Retain
**Equity**	Inter-facility Disparity index	3.8	0.79	Retain

Moreover, 18 KPIs were removed or merged due to redundancy, limited feasibility, or lack of standardized measurement in community and retail pharmacy settings. These included advanced digital indicators, complex equity measures, and sub-indicators overlapping with broader KPI constructs as given in Table 4.

**Table 4:** KPIs removed or merged after Delphi Round 1 (n = 18).

KPI Category	Removed / Merged KPIs	Action	Reason
**Workforce**	Pharmacist workload index	Merged	Redundant with pharmacist-to-patient ratio
	Technician workload distribution	Merged	Covered under workforce equity indicator
	Staffing adequacy score	Removed	Overlap with workforce indicators
**Quality**	Patient satisfaction index	Removed	Lack of standardized measurement in pharmacies
	Clinical intervention documentation rate	Removed	Low feasibility in routine practice
**Safety**	ADR reporting rate	Merged	Included under medication error reporting
	Near-miss reporting frequency	Removed	Underreporting in community/retail settings
**Availability**	Order fulfillment time	Merged	Covered under stock-out indicators
	Procurement delay index	Removed	External system dependency
**Equity**	Gender-based access difference	Removed	Data not routinely captured
	Socioeconomic access index	Removed	Measurement inconsistency
	Geographic accessibility score	Merged	Incorporated into disparity index
	Urban–rural pricing variation	Removed	Data heterogeneity
**Digital**	Electronic prescription adoption rate	Removed	Low E-prescribing penetration
	Digital record completeness	Removed	Predominantly manual systems
	Barcode scanning compliance	Removed	Not implemented in most settings

### Delphi Round 2: Final KPI consensus

Following iterative refinement, 11 KPIs were finalized across six domains: workforce, efficiency, quality, safety, availability, and equity. Indicators such as pharmacist-to-patient ratio (mean = 4.8, I-CVI = 0.92), stock-out frequency (mean = 4.6, I-CVI = 0.96), and essential medicine availability (mean = 4.5, I-CVI = 0.89) demonstrated strong expert agreement. The inclusion of the inter-facility disparity index reflects the study’s emphasis on equity measurement as mentioned in Table 5.

**Table 5:** Delphi Round 2 final KPI consensus (final set, N = 11 KPIs).

KPI	Domain	Mean Rating	I-CVI	Decision
**Pharmacist-to-patient ratio**	Workforce	4.8	0.92	Finalized
**Workforce equity ratio**	Equity	4.4	0.86	Finalized
**Patient waiting time**	Efficiency	4.2	0.83	Finalized
**Average dispensing time**	Efficiency	4.2	0.88	Finalized
**Counseling rate (%)**	Quality	4.3	0.83	Finalized
**Documentation completeness**	Quality	4.1	0.80	Finalized
**Medication error reporting**	Safety	3.9	0.78	Finalized
**Stock-out frequency**	Availability	4.6	0.96	Finalized
**Stock-out duration**	Availability	4.4	0.92	Finalized
**Essential medicine availability**	Availability	4.5	0.89	Finalized
**Inter-facility disparity index**	Equity	4.1	0.79	Finalized

### Final KPI framework for dashboard

The finalized KPIs were operationalized into a structured framework, with clearly defined formulas and measurement approaches. These indicators collectively capture workforce capacity, service efficiency, quality of care, patient safety, medicine availability, and equity in service provision. This framework formed the basis for dashboard development and real-time performance monitoring as shown in Table 6.

**Table 6:** Final KPI framework for dashboard implementation.

KPI	Domain	Formula / Measurement	Purpose
**Pharmacist-to-patient ratio**	Workforce	Patients ÷ Pharmacists	Workforce capacity
**Workforce equity ratio**	Equity	Rural vs Urban staffing ratio	Inequality measurement
**Average dispensing time**	Efficiency	Minutes per patient	Workflow efficiency
**Patient waiting time**	Efficiency	Queue time	Access efficiency
**Counseling rate (%)**	Quality	% patients counseled	Quality of care
**Documentation completeness**	Quality	% complete records	Accountability
**Medication error reporting**	Safety	Errors per month	Patient safety
**Stock-out frequency**	Availability	Episodes/month	Supply chain reliability
**Stock-out duration**	Availability	Days/month	Severity of shortage
**Essential medicine availability**	Availability	% availability	Access to medicines
**Disparity index**	Equity	Urban ÷ Rural KPI	Equity measurement

### Dashboard design and features

The developed dashboard incorporated multiple functional components as given in Table 7, including KPI summary cards, interactive filters, dynamic visualizations, and an equity comparison panel. These features enable users to monitor performance trends, compare urban and rural settings, and identify areas requiring intervention. Automated alert systems further enhance decision-making by highlighting underperforming indicators.

**Table 7:** Dashboard components and features.

Component	Description	Function
KPI Cards	At top of dashboard	Quick summary indicators
Filters	Hospital type, district, month	Comparison and deep dives
Bar charts	Counselling, dispensing time	Visual comparisons
Line graphs	Stock-out trends	Monitor over time
Gauge charts	KPI thresholds	Instant performance check
Equity panel	Disparity index visualization	Shows inequity
Alert system	Automatic color flagging	Highlights low performance

### Usability evaluation

Usability testing demonstrated high acceptability of the dashboard among all user groups as mentioned in Table 8. The overall System Usability Scale (SUS) score was 82.5 ± 6.1, indicating excellent usability. Community pharmacists reported the highest usability scores (84.1 ± 5.8), followed by administrators and technicians. These findings suggest that the dashboard is intuitive and suitable for routine use in pharmacy settings.

**Table 8:** System usability scale (SUS) results.

Group	n	Mean SUS	SD	Result implication
**Community Pharmacists**	10	84.1	5.8	Excellent usability
**Pharmacy Technicians**	6	80.3	6.9	Good usability
**Administrators**	4	82.0	6.2	Excellent usability
**Overall**	20	82.5	6.1	High usability (Acceptable–Excellent range)

### Subgroup comparison by profession

Table 9 summarizes subgroup differences across professional roles in terms of usability, KPI appraisal, and qualitative focus. SUS scores were consistently high across all groups, with pharmacists reporting slightly higher usability than administrators and technicians. Delphi KPI ratings were derived from a unified expert panel and therefore were not stratified by profession. Qualitative findings indicated role-specific emphases, with pharmacists focusing on clinical workflow and patient counselling, technicians on dispensing and operational workload, and administrators on system-level resource and staffing management.

**Table 9:** Subgroup comparison by profession.

Measure	Pharmacists	Technicians	Administrators
**SUS Score (mean ± SD)**	84.1 ± 5.8	80.3 ± 6.9	82.0 ± 6.2
**Mean KPI Rating (Delphi)**	Not stratified by profession (panel-level consensus ratings applied uniformly across expert group)	Not stratified by profession	Not stratified by profession
**Key Themes (qualitative emphasis)**	Greater focus on clinical workflow constraints, counselling processes, and patient-facing service delivery issues	Emphasis on dispensing operations, workload distribution, and medication handling bottlenecks	Focus on system-level management, staffing allocation, and resource constraints

### Qualitative usability feedback

Qualitative feedback reinforced the quantitative usability findings as mentioned in Table 10. Participants highlighted ease of navigation and visual clarity as key strengths, particularly the use of color-coded indicators. The dashboard was also perceived as a valuable decision-support tool for identifying rural service gaps. However, respondents emphasized the need for training, especially for staff in rural settings.

**Table 10:** Qualitative usability themes.

Theme	Supporting Feedback	Analytical interpretation
Ease of Navigation	“Simple and clear”	User-friendly
Visual Clarity	“Color coding is very helpful”	Good design quality
Decision Support	“Shows exactly where rural gaps exist”	Useful for managers
Training Need	“Rural staff need more sessions”	Capacity building needed

### Content validity of KPIs

Content validity analysis demonstrated strong agreement among experts, with an overall S-CVI/Ave of 0.89, indicating excellent validity of the KPI framework. Individual indicators showed high I-CVI values, particularly for stock-out duration (0.96) and pharmacist-to-patient ratio (0.92), confirming their relevance and applicability as shown in Table 11.

**Table 11:** Content validity index (CVI).

KPI	I-CVI
Pharmacist-to-patient ratio	0.92
Dispensing time	0.88
Stock-out duration	0.96
Counseling rate (%)	0.83
Documentation completeness	0.80
Disparity index	0.79
Workforce equity ratio	0.86
Essential medicine availability	0.89
Medication error reporting	0.78
S-CVI/Ave	0.89

### Equity analysis: Urban vs rural comparison

Significant disparities were observed between urban and rural community pharmacies across multiple indicators as shown in Table 12. Rural settings showed higher stock-out duration (13.1 vs 6.2 days), lower counselling rates (55% vs 78%), and poorer pharmacist-to-patient ratios (1:120 vs 1:65). Documentation completeness and essential medicine availability were also lower in rural pharmacies. These findings highlight substantial inequities in pharmaceutical service delivery.

**Table 12:** Equity indicator outcomes (urban vs rural community pharmacies).

Indicator	Urban Mean	Rural Mean	Equity Ratio (Urban/Rural)	Result implication
**Stock-out duration (days)**	6.2	13.1	2.1	Rural disadvantage
**Counseling rate (%)**	78%	55%	1.42	Lower rural quality
**Pharmacist-to-patient ratio**	1:65	1:120	1.85	Rural understaffing
**Documentation completeness**	84%	61%	1.38	Rural deficit
**Essential medicine availability (%)**	92%	74%	1.24	Rural inequity

### System performance evaluation

Dashboard demonstrated strong technical performance as mentioned in Table 13, with optimal data refresh rates, fast load times (1.8 seconds), and zero system errors. User interaction metrics indicated efficient navigation, requiring an average of only two clicks to access core KPIs. High mobile responsiveness further supports its applicability in diverse practice settings.

**Table 13:** System performance metrics for dashboard.

Performance Area	Metric	Value	Status
Data refresh rate	Auto-refresh	60 sec	Optimal
Load time	Page load	1.8 sec	Excellent
Error logs	Failures	0	Stable
User clicks to core KPI	Average	2 clicks	Good navigation
Mobile responsiveness	Score	92%	Very good

## Discussion

This study developed an equity-focused KPI dashboard for community pharmacies in Pakistan using Delphi consensus and usability testing. Eleven KPIs across six domains were finalized, highlighting workforce shortages, rural medicine stock-outs, poor counselling rates, and operational inefficiencies. Strong urban–rural disparities were evident, particularly in stock-out duration and pharmacist availability. Content validity was high (S-CVI/Ave = 0.89), and usability testing showed excellent acceptability (SUS = 82.5). Overall, the dashboard demonstrated feasibility as a structured tool for monitoring pharmacy performance and equity in resource-limited settings. The findings of this study align strongly with existing global literature emphasizing persistent inequities in community pharmacy services, particularly in low- and middle-income countries (LMICs) [22–24]. Workforce maldistribution observed in this study, with significantly lower pharmacist-to-patient ratios in rural areas, reflects similar patterns reported by the International Pharmaceutical Federation (FIP) [25] and World Health Organization (WHO) report [26], where rural areas consistently face shortages of trained pharmacy personnel. These structural imbalances have been associated with reduced patient counseling time and compromised pharmaceutical care quality.

Medicine availability issues identified in this study, including frequent stock-outs and longer stock-out durations in rural pharmacies, are also consistent with evidence from Kenya, Bangladesh, and Pakistan, where fragmented supply chains and weak inventory systems limit access to essential medicines [27–29]. The magnitude of rural-urban disparity observed reinforces prior findings that supply chain inefficiencies disproportionately affect underserved regions. Whereas, quality-related gaps, particularly low counseling rates and incomplete documentation, mirror studies from South Asia that link poor pharmacy service quality to high workload and lack of standardized protocols [30]. Similarly, operational inefficiencies such as prolonged dispensing times and reliance on manual systems reflect broader LMIC health system limitations in digital infrastructure adoption [31].

Importantly, this study extends existing literature by integrating equity as a measurable KPI dimension within a real-time dashboard. While most prior frameworks focus on efficiency and quality, the inclusion of disparity indices provides a more holistic health system perspective [32–33]. High usability findings further align with global digital health evidence demonstrating that intuitive system design facilitates adoption within clinical workflows. Collectively, the results reinforce established systemic challenges in pharmacy practice while advancing an equity-oriented, data-driven approach to performance monitoring in pharmacy systems. However, implementation of the proposed dashboard in low-resource rural settings may be constrained by limited digital infrastructure, workforce shortages, and inconsistent internet connectivity [34]. These constraints may affect real-time data entry and system responsiveness in peripheral pharmacies. However, the use of a lightweight platform such as Microsoft Power BI, combined with standardized KPI reporting, provides a scalable and cost-effective approach [16] that can function within existing resource limitations. Long-term sustainability will depend on integration with provincial pharmacy regulatory frameworks, establishment of routine data reporting mechanisms [35], and continuous stakeholder engagement. Strengthening governance structures, particularly those related to data quality assurance, performance monitoring, and accountability, will be essential to ensure sustained adoption and meaningful use of the dashboard in routine pharmacy practice.

A key strength of this study is the rigorous development of an equity-oriented KPI dashboard using a mixed-methods design, combining qualitative needs assessment with Delphi consensus and validated through CVI and SUS, ensuring methodological robustness and contextual relevance. The explicit inclusion of equity indicators extends conventional pharmacy performance frameworks. However, limitations include reliance on structured rather than real-time operational data, a geographically limited sample, and small usability testing cohort, which may affect generalizability. Despite this, the dashboard offers a scalable decision-support tool for LMICs to improve pharmacy performance, address rural-urban disparities, and support data-driven health system strengthening.

## Conclusion

This study developed and validated a comprehensive KPI dashboard for community and retail pharmacy settings in Pakistan, revealing significant urban-rural disparities in workforce distribution, medicine availability, service quality, and operational efficiency. The finalized 11-KPI framework, supported by strong expert consensus and high usability scores, provides a practical tool for real-time performance monitoring and equity assessment. Its integration of multidimensional indicators makes it a scalable solution for improving pharmacy services. The dashboard holds strong potential to support data-driven decision-making and strengthen equitable pharmaceutical care in LMIC health systems.

## Abbreviations

KPI: Key Performance Indicator
CVI: Content Validity Index
S-CVI: Scale Content Validity Index
I-CVI: Item Content Validity Index
SUS: System Usability Scale
LMIC: Low- and Middle-Income Countries
Power BI: Microsoft Power Business Intelligence
DAX: Data Analysis Expressions
WHO: World Health Organization
FIP: International Pharmaceutical Federation
UHC: Universal Health Coverage
