# Supplementary material for: Design and validation of a key performance indicator dashboard for equity in community and retail pharmacy services in Pakistan
Source: PLoS One. 2026 Jul 10;21(7):e0349972. doi: 10.1371/journal.pone.0349972 (PMC13353981; doi:10.1371/journal.pone.0349972)
Supplement: Attachment — Submitted filename: Response to Reviewers (13-06-26).docx [file pone.0349972.s002.docx]

**Response to Reviewers**

We sincerely thank the Academic Editor and Reviewers for their careful evaluation and constructive feedback. We have revised the manuscript accordingly. All comments have been addressed in detail as outlined below.

**Academic Editor Comments**

**Comment 1:** Introduction requires more critical synthesis, comparison with KPI frameworks, and Pakistan-specific evidence

**Answer:** We appreciate this important suggestion. The Introduction has been revised to include a more critical synthesis of existing pharmacy KPI frameworks, including cpKPIs and PCNE indicators. We further highlighted their limitations in low- and middle-income countries, particularly regarding workforce shortages, fragmented supply chains, and weak digital infrastructure. In addition, we incorporated Pakistan-specific evidence describing disparities in pharmacist distribution, rural regulatory gaps, and infrastructure constraints, thereby strengthening the contextual and analytical depth of the study.

**Comment 2:** Methods require additional rigor, sample size rationale, and dashboard architecture description

**Answer:** We have strengthened the Methods section by enhancing the rigor of qualitative analysis. This includes independent coding by two researchers, iterative codebook development, and investigator triangulation. A clear justification for sample size has been added based on the principle of thematic saturation, with recruitment continuing until no new themes emerged. Furthermore, a detailed technical description of the dashboard architecture has been included, outlining data input sources, processing workflow, KPI computation layer, and visualization framework to improve methodological transparency.

**Comment 3:** Clarification of whether data are real, pilot, or hypothetical

**Answer:** We have clarified this point in the Results section. The dashboard was developed and tested using pilot operational datasets obtained from participating community and retail pharmacies. No simulated or hypothetical data were used at any stage of analysis.

**Comment 4:** Discussion should include implementation feasibility, sustainability, and governance

**Answer:** A dedicated paragraph has been added to the Discussion addressing implementation feasibility in low-resource rural settings. This includes challenges related to digital infrastructure, workforce constraints, and internet connectivity. We also discuss sustainability considerations such as integration with provincial regulatory systems, routine reporting mechanisms, stakeholder engagement, and governance structures for data quality assurance and accountability.

**Reviewer #1 Comments**

**Comment 1:** Add theoretical/model framework (Gustafson et al., 2023)

**Answer:** We thank the reviewer for this valuable suggestion. The Introduction has been strengthened by incorporating an equity-focused implementation framework informed by Gustafson et al. (2023). This framework provides theoretical grounding for the four domains used in the study, workforce capacity, medicine availability, service quality, and access equity, thereby strengthening the conceptual basis of the KPI dashboard.

**Comment 2:** Methods - respondent selection, response rate, and data collection clarity

**Answer:** We have revised the Methods section to provide a clearer description of participant selection using purposive sampling. Additional details on recruitment procedures, eligibility criteria, and data collection methods have been included. The response rate (82.4%) has also been reported to enhance transparency.

**Comment 3:** Results - equity analysis between professions

**Answer:** We have addressed this comment by introducing a dedicated subgroup analysis. This includes comparison of SUS scores and qualitative thematic differences across pharmacists, pharmacy technicians, and administrators. A new table has also been added summarizing professional differences in usability perception and thematic emphasis, while Delphi KPI ratings were appropriately reported at the consensus panel level.

**Reviewer #2 Comments**

**Comment 1:** KPI relevance and scalability – inclusion of registered pharmacist indicator

**Answer:** We acknowledge this important suggestion. The role of registered pharmacists is inherently captured within the workforce-related KPIs, particularly the pharmacist-to-patient ratio and workforce equity indicators. These metrics directly reflect pharmacist availability in community pharmacy settings and have been emphasized in the revised Discussion as key scalability indicators for LMIC contexts.

**Comment 2:** Data verification and validation of self-reported data

**Answer:** We have added a new subsection describing data verification procedures. Operational data were validated through cross-checking with pharmacy inventory records, dispensing logs, and administrative documentation prior to integration into the dashboard. This multi-source validation approach was used to enhance data reliability and reduce reporting bias.
