# Supplementary material for: Design and validation of a key performance indicator dashboard for equity in community and retail pharmacy services in Pakistan
Source: PLoS One. 2026 Jul 10;21(7):e0349972. doi: 10.1371/journal.pone.0349972 (PMC13353981; doi:10.1371/journal.pone.0349972)
Supplement: Peer review history

# Peer review: Decision Letter 0

**DOI:** 10.1371/journal.pone.0349972.r001

**Authors:** Ibrahim Jahun

28 May 2026

PONE-D-26-22477

Design and Validation of a Key Performance Indicator Dashboard for Equity in Community and Retail Pharmacy Services in Pakistan

PLOS One

Dear Dr. Hammad,

Thank you for submitting your manuscript to PLOS ONE. After careful consideration, we feel that it has merit but does not fully meet PLOS ONE’s publication criteria as it currently stands. Therefore, we invite you to submit a revised version of the manuscript that addresses the points raised during the review process.

**ACADEMIC EDITOR:**

The paper addresses an important and timely issue in pharmacy system strengthening in low- and middle-income countries (LMICs), particularly the lack of equity-focused monitoring tools for community and retail pharmacy services. The paper is well written but will require some minor improvement regarding methodological, analytical, and reporting limitations as described in my comments below and by the 2 reviewers.

**Introduction:**  The section provides good contextual background on pharmacy inequities in LMICs and Pakistan. However, it sounds more descriptive and may require some critical synthesis. Specifically, there are needs for critical comparison with existing pharmacy KPI frameworks and why current system fails in LMICs.  Also include more Pakistan-specific evidence on pharmacy infrastructure disparities.

**Methods:**  Multi-phase methodological structure and Delphi consensus are appropriate for dashboard development. The section will benefit from additional methodological rigor to qualitative analysis. Also include sample size rational and add technical description of the dashboard architecture.

**Results:**  The section is appropriate. However, it is unclear whether operational pharmacy data are real-world, pilot or hypothetical data.

**Discussion:**  It will be good to discuss implementation feasibility in low-resource rural settings including sustainability and governance considerations.

Please submit your revised manuscript by Jun 27 2026 11:59PM. If you will need more time than this to complete your revisions, please reply to this message or contact the journal office at plosone@plos.org. When you're ready to submit your revision, log on to https://www.editorialmanager.com/pone/ and select the 'Submissions Needing Revision' folder to locate your manuscript file.

Please include the following items when submitting your revised manuscript:

- A letter that responds to each point raised by the academic editor and reviewer(s). You should upload this letter as a separate file labeled 'Response to Reviewers'.
- A marked-up copy of your manuscript that highlights changes made to the original version. You should upload this as a separate file labeled 'Revised Manuscript with Track Changes'.
- An unmarked version of your revised paper without tracked changes. You should upload this as a separate file labeled 'Manuscript'.

If you would like to make changes to your financial disclosure, please include your updated statement in your cover letter. Guidelines for resubmitting your figure files are available below the reviewer comments at the end of this letter.

As the corresponding author, your ORCID iD is verified in the submission system and will appear in the published article. PLOS supports the use of ORCID, and we encourage all coauthors to register for an ORCID iD and use it as well. Please encourage your coauthors to verify their ORCID iD within the submission system before final acceptance, as unverified ORCID iDs will not appear in the published article. *Only*  the individual author can complete the verification step; PLOS staff *cannot*  verify ORCID iDs on behalf of authors.

We look forward to receiving your revised manuscript.

Kind regards,

Ibrahim Jahun, MD, MSC, PhD

Academic Editor

PLOS One

Journal Requirements:

When submitting your revision, we need you to address these additional requirements.

1. Please ensure that your manuscript meets PLOS ONE's style requirements, including those for file naming. The PLOS ONE style templates can be found at

https://journals.plos.org/plosone/s/file?id=wjVg/PLOSOne_formatting_sample_main_body.pdf and

https://journals.plos.org/plosone/s/file?id=ba62/PLOSOne_formatting_sample_title_authors_affiliations.pdf

2. In the online submission form, you indicated that datasets generated and/or analyzed during the current study are available from the corresponding author on reasonable request.

All PLOS journals now require all data underlying the findings described in their manuscript to be freely available to other researchers, either 1. In a public repository, 2. Within the manuscript itself, or 3. Uploaded as supplementary information.

This policy applies to all data except where public deposition would breach compliance with the protocol approved by your research ethics board. If your data cannot be made publicly available for ethical or legal reasons (e.g., public availability would compromise patient privacy), please explain your reasons on resubmission and your exemption request will be escalated for approval.

3. We note that you have indicated that there are restrictions to data sharing for this study. For studies involving human research participant data or other sensitive data, we encourage authors to share de-identified or anonymized data. However, when data cannot be publicly shared for ethical reasons, we allow authors to make their data sets available upon request. For information on unacceptable data access restrictions, please see http://journals.plos.org/plosone/s/data-availability#loc-unacceptable-data-access-restrictions.

Before we proceed with your manuscript, please address the following prompts:

a) If there are ethical or legal restrictions on sharing a de-identified data set, please explain them in detail (e.g., data contain potentially identifying or sensitive patient information, data are owned by a third-party organization, etc.) and who has imposed them (e.g., a Research Ethics Committee or Institutional Review Board, etc.). Please also provide contact information for a data access committee, ethics committee, or other institutional body to which data requests may be sent.

b) If there are no restrictions, please upload the minimal anonymized data set necessary to replicate your study findings to a stable, public repository and provide us with the relevant URLs, DOIs, or accession numbers. Please see http://www.bmj.com/content/340/bmj.c181.long for guidelines on how to de-identify and prepare clinical data for publication. For a list of recommended repositories, please see https://journals.plos.org/plosone/s/recommended-repositories. You also have the option of uploading the data as Supporting Information files, but we would recommend depositing data directly to a data repository if possible.

Please update your Data Availability statement in the submission form accordingly.

4. Your ethics statement should only appear in the Methods section of your manuscript. If your ethics statement is written in any section besides the Methods, please delete it from any other section.

5. If the reviewer comments include a recommendation to cite specific previously published works, please review and evaluate these publications to determine whether they are relevant and should be cited. There is no requirement to cite these works unless the editor has indicated otherwise.

6. Please review your reference list to ensure that it is complete and correct. If you have cited papers that have been retracted, please include the rationale for doing so in the manuscript text, or remove these references and replace them with relevant current references. Any changes to the reference list should be mentioned in the rebuttal letter that accompanies your revised manuscript. If you need to cite a retracted article, indicate the article’s retracted status in the References list and also include a citation and full reference for the retraction notice.

Reviewers' comments:

Reviewer's Responses to Questions

**Comments to the Author**

1. Is the manuscript technically sound, and do the data support the conclusions?

The manuscript must describe a technically sound piece of scientific research with data that supports the conclusions. Experiments must have been conducted rigorously, with appropriate controls, replication, and sample sizes. The conclusions must be drawn appropriately based on the data presented.

Reviewer #1: Yes

Reviewer #2: Partly

**********

2. Has the statistical analysis been performed appropriately and rigorously?

Reviewer #1: Yes

Reviewer #2: Yes

**********

3. Have the authors made all data underlying the findings in their manuscript fully available?

The PLOS Data policy requires authors to make all data underlying the findings described in their manuscript fully available without restriction, with rare exception (please refer to the Data Availability Statement in the manuscript PDF file). The data should be provided as part of the manuscript or its supporting information, or deposited to a public repository. For example, in addition to summary statistics, the data points behind means, medians and variance measures should be available. If there are restrictions on publicly sharing data—e.g. participant privacy or use of data from a third party—those must be specified.

Reviewer #1: Yes

Reviewer #2: Yes

**********

4. Is the manuscript presented in an intelligible fashion and written in standard English?

PLOS ONE does not copyedit accepted manuscripts, so the language in submitted articles must be clear, correct, and unambiguous. Any typographical or grammatical errors should be corrected at revision, so please note any specific errors here.

Reviewer #1: Yes

Reviewer #2: Yes

**********

5. Review Comments to the Author

Please use the space provided to explain your answers to the questions above. You may also include additional comments for the author, including concerns about dual publication, research ethics, or publication ethics. (Please upload your review as an attachment if it exceeds 20,000 characters)

Reviewer #1: Introduction and Discussion

The author should add theory or model to explain the association between variables (Gustafson, 2023) – four domains (page 11).

Reference:

Gustafson, P., Abdul Aziz, Y., Lambert, M. et al. A scoping review of equity-focused implementation theories, models and frameworks in healthcare and their application in addressing ethnicity-related health inequities. Implementation Sci 18, 51 (2023). https://doi.org/10.1186/s13012-023-01304-0

Methods

How does the author determine or choose the respondents? What is the response rate? How does the author collect the data?

Results

What is the equity analysis between professions? Community Pharmacists, Pharmacy Technicians, Pharmacy Administrators have different roles and responsibilities.

Reviewer #2: Its a good initiative, much needed in LMIC. needs to be scaled up appropriately. One of the KPIs when you want to scale up is i believe should have been presence of registered pharmacist in community pharmacy.

My question this seems to be self reported data by pharmacists, staff and administrators. How was it verified? and validated? Or was it digitally monitored if pharmacies have full computerized system.

**********

6. PLOS authors have the option to publish the peer review history of their article (what does this mean?). If published, this will include your full peer review and any attached files.

If you choose “no”, your identity will remain anonymous but your review may still be made public.

**Do you want your identity to be public for this peer review?**  For information about this choice, including consent withdrawal, please see our Privacy Policy.

Reviewer #1: No

Reviewer #2: No

**********

[NOTE: If reviewer comments were submitted as an attachment file, they will be attached to this email and accessible via the submission site. Please log into your account, locate the manuscript record, and check for the action link "View Attachments". If this link does not appear, there are no attachment files.]

To ensure your figures meet our technical requirements, please review our figure guidelines: https://journals.plos.org/plosone/s/figures

You may also use PLOS’s free figure tool, NAAS, to help you prepare publication quality figures: https://journals.plos.org/plosone/s/figures#loc-tools-for-figure-preparation.

NAAS will assess whether your figures meet our technical requirements by comparing each figure against our figure specifications.

# Author comment: Author response to Decision Letter 1

**DOI:** 10.1371/journal.pone.0349972.r002

16 Jun 2026

Response to Reviewers

We sincerely thank the Academic Editor and Reviewers for their careful evaluation and constructive feedback. We have revised the manuscript accordingly. All comments have been addressed in detail as outlined below.

Academic Editor Comments

Comment 1: Introduction requires more critical synthesis, comparison with KPI frameworks, and Pakistan-specific evidence

Answer: We appreciate this important suggestion. The Introduction has been revised to include a more critical synthesis of existing pharmacy KPI frameworks, including cpKPIs and PCNE indicators. We further highlighted their limitations in low- and middle-income countries, particularly regarding workforce shortages, fragmented supply chains, and weak digital infrastructure. In addition, we incorporated Pakistan-specific evidence describing disparities in pharmacist distribution, rural regulatory gaps, and infrastructure constraints, thereby strengthening the contextual and analytical depth of the study.

Comment 2: Methods require additional rigor, sample size rationale, and dashboard architecture description

Answer: We have strengthened the Methods section by enhancing the rigor of qualitative analysis. This includes independent coding by two researchers, iterative codebook development, and investigator triangulation. A clear justification for sample size has been added based on the principle of thematic saturation, with recruitment continuing until no new themes emerged. Furthermore, a detailed technical description of the dashboard architecture has been included, outlining data input sources, processing workflow, KPI computation layer, and visualization framework to improve methodological transparency.

Comment 3: Clarification of whether data are real, pilot, or hypothetical

Answer: We have clarified this point in the Results section. The dashboard was developed and tested using pilot operational datasets obtained from participating community and retail pharmacies. No simulated or hypothetical data were used at any stage of analysis.

Comment 4: Discussion should include implementation feasibility, sustainability, and governance

Answer: A dedicated paragraph has been added to the Discussion addressing implementation feasibility in low-resource rural settings. This includes challenges related to digital infrastructure, workforce constraints, and internet connectivity. We also discuss sustainability considerations such as integration with provincial regulatory systems, routine reporting mechanisms, stakeholder engagement, and governance structures for data quality assurance and accountability.

Reviewer #1 Comments

Comment 1: Add theoretical/model framework (Gustafson et al., 2023)

Answer: We thank the reviewer for this valuable suggestion. The Introduction has been strengthened by incorporating an equity-focused implementation framework informed by Gustafson et al. (2023). This framework provides theoretical grounding for the four domains used in the study, workforce capacity, medicine availability, service quality, and access equity, thereby strengthening the conceptual basis of the KPI dashboard.

Comment 2: Methods - respondent selection, response rate, and data collection clarity

Answer: We have revised the Methods section to provide a clearer description of participant selection using purposive sampling. Additional details on recruitment procedures, eligibility criteria, and data collection methods have been included. The response rate (82.4%) has also been reported to enhance transparency.

Comment 3: Results - equity analysis between professions

Answer: We have addressed this comment by introducing a dedicated subgroup analysis. This includes comparison of SUS scores and qualitative thematic differences across pharmacists, pharmacy technicians, and administrators. A new table has also been added summarizing professional differences in usability perception and thematic emphasis, while Delphi KPI ratings were appropriately reported at the consensus panel level.

Reviewer #2 Comments

Comment 1: KPI relevance and scalability – inclusion of registered pharmacist indicator

Answer: We acknowledge this important suggestion. The role of registered pharmacists is inherently captured within the workforce-related KPIs, particularly the pharmacist-to-patient ratio and workforce equity indicators. These metrics directly reflect pharmacist availability in community pharmacy settings and have been emphasized in the revised Discussion as key scalability indicators for LMIC contexts.

Comment 2: Data verification and validation of self-reported data

Answer: We have added a new subsection describing data verification procedures. Operational data were validated through cross-checking with pharmacy inventory records, dispensing logs, and administrative documentation prior to integration into the dashboard. This multi-source validation approach was used to enhance data reliability and reduce reporting bias.

Attachment Submitted filename: Response to Reviewers (13-06-26).docx

# Peer review: Decision Letter 1

**DOI:** 10.1371/journal.pone.0349972.r003

**Authors:** Ibrahim Jahun, Ibrahim Jahun

25 Jun 2026

Design and Validation of a Key Performance Indicator Dashboard for Equity in Community and Retail Pharmacy Services in Pakistan

PONE-D-26-22477R1

Dear Dr. Hammad,

We’re pleased to inform you that your manuscript has been judged scientifically suitable for publication and will be formally accepted for publication once it meets all outstanding technical requirements.

Within one week, you’ll receive an e-mail detailing the required amendments. When these have been addressed, you’ll receive a formal acceptance letter and your manuscript will be scheduled for publication.

An invoice will be generated when your article is formally accepted. Please note, if your institution has a publishing partnership with PLOS and your article meets the relevant criteria, all or part of your publication costs will be covered. Please make sure your user information is up-to-date by logging into Editorial Manager at Editorial Manager® and clicking the ‘Update My Information' link at the top of the page. For questions related to billing, please contact billing support.

If your institution or institutions have a press office, please notify them about your upcoming paper to help maximize its impact. If they’ll be preparing press materials, please inform our press team as soon as possible -- no later than 48 hours after receiving the formal acceptance. Your manuscript will remain under strict press embargo until 2 pm Eastern Time on the date of publication. For more information, please contact onepress@plos.org.

Kind regards,

Ibrahim Jahun, MD, MSC, PhD

Academic Editor

PLOS One

Additional Editor Comments (optional):

Reviewers' comments:

Reviewer's Responses to Questions

**Comments to the Author**

1. If the authors have adequately addressed your comments raised in a previous round of review and you feel that this manuscript is now acceptable for publication, you may indicate that here to bypass the “Comments to the Author” section, enter your conflict of interest statement in the “Confidential to Editor” section, and submit your "Accept" recommendation.

Reviewer #1: (No Response)

Reviewer #2: (No Response)

**********

2. Is the manuscript technically sound, and do the data support the conclusions?

The manuscript must describe a technically sound piece of scientific research with data that supports the conclusions. Experiments must have been conducted rigorously, with appropriate controls, replication, and sample sizes. The conclusions must be drawn appropriately based on the data presented.

Reviewer #1: (No Response)

Reviewer #2: Yes

**********

3. Has the statistical analysis been performed appropriately and rigorously?

Reviewer #1: (No Response)

Reviewer #2: Yes

**********

4. Have the authors made all data underlying the findings in their manuscript fully available?

The PLOS Data policy requires authors to make all data underlying the findings described in their manuscript fully available without restriction, with rare exception (please refer to the Data Availability Statement in the manuscript PDF file). The data should be provided as part of the manuscript or its supporting information, or deposited to a public repository. For example, in addition to summary statistics, the data points behind means, medians and variance measures should be available. If there are restrictions on publicly sharing data—e.g. participant privacy or use of data from a third party—those must be specified.

Reviewer #1: (No Response)

Reviewer #2: Yes

**********

5. Is the manuscript presented in an intelligible fashion and written in standard English?

PLOS ONE does not copyedit accepted manuscripts, so the language in submitted articles must be clear, correct, and unambiguous. Any typographical or grammatical errors should be corrected at revision, so please note any specific errors here.

Reviewer #1: (No Response)

Reviewer #2: Yes

**********

6. Review Comments to the Author

Please use the space provided to explain your answers to the questions above. You may also include additional comments for the author, including concerns about dual publication, research ethics, or publication ethics. (Please upload your review as an attachment if it exceeds 20,000 characters)

Reviewer #1: (No Response)

Reviewer #2: (No Response)

**********

7. PLOS authors have the option to publish the peer review history of their article (what does this mean?). If published, this will include your full peer review and any attached files.

If you choose “no”, your identity will remain anonymous but your review may still be made public.

**Do you want your identity to be public for this peer review?**  For information about this choice, including consent withdrawal, please see our Privacy Policy.

Reviewer #1: No

Reviewer #2: No

**********

# Editor report: Acceptance letter

**DOI:** 10.1371/journal.pone.0349972.r004

**Authors:** Ibrahim Jahun, Ibrahim Jahun

PONE-D-26-22477R1

PLOS One

Dear Dr. Hammad,

I'm pleased to inform you that your manuscript has been deemed suitable for publication in PLOS One. Congratulations! Your manuscript is now being handed over to our production team.

At this stage, our production department will prepare your paper for publication. This includes ensuring the following:

* All references, tables, and figures are properly cited

* All relevant supporting information is included in the manuscript submission,

* There are no issues that prevent the paper from being properly typeset

You will receive further instructions from the production team, including instructions on how to review your proof when it is ready. Please keep in mind that we are working through a large volume of accepted articles, so please give us a few days to review your paper and let you know the next and final steps.

Lastly, if your institution or institutions have a press office, please let them know about your upcoming paper now to help maximize its impact. If they'll be preparing press materials, please inform our press team within the next 48 hours. Your manuscript will remain under strict press embargo until 2 pm Eastern Time on the date of publication. For more information, please contact onepress@plos.org.

You will receive an invoice from PLOS for your publication fee after your manuscript has reached the completed accept phase. If you receive an email requesting payment before acceptance or for any other service, this may be a phishing scheme. Learn how to identify phishing emails and protect your accounts at https://explore.plos.org/phishing.

If we can help with anything else, please email us at customercare@plos.org.

Thank you for submitting your work to PLOS ONE and supporting open access.

Kind regards,

PLOS ONE Editorial Office Staff

on behalf of

Dr. Ibrahim Jahun

Academic Editor

PLOS One
